# Cauchy non-convex sparse feature selection method for the high-dimensional small-sample problem in motor imagery EEG decoding

**DOI:** 10.3389/fnins.2023.1292724

**Published:** 2023-11-03

**Authors:** Shaorong Zhang, Qihui Wang, Benxin Zhang, Zhen Liang, Li Zhang, Linling Li, Gan Huang, Zhiguo Zhang, Bao Feng, Tianyou Yu

**Affiliations:** ^1^Guangdong Key Laboratory for Biomedical Measurements and Ultrasound Imaging, National-Regional Key Technology Engineering Laboratory for Medical Ultrasound, School of Biomedical Engineering, Shenzhen University Medical School, Shenzhen, China; ^2^School of Electronic Information and Automation, Guilin University of Aerospace Technology, Guilin, China; ^3^School of Electronic Engineering and Automation, Guilin University of Electronic Technology, Guilin, China; ^4^Institute of Computing and Intelligence, Harbin Institute of Technology, Shenzhen, China; ^5^School of Automation Science and Engineering, South China University of Technology, Guangzhou, China

**Keywords:** motor imagery, EEG decoding, feature selection, nonconvex regularization, high-dimensional small-sample

## Abstract

**Introduction:**

The time, frequency, and space information of electroencephalogram (EEG) signals is crucial for motor imagery decoding. However, these temporal-frequency-spatial features are high-dimensional small-sample data, which poses significant challenges for motor imagery decoding. Sparse regularization is an effective method for addressing this issue. However, the most commonly employed sparse regularization models in motor imagery decoding, such as the least absolute shrinkage and selection operator (LASSO), is a biased estimation method and leads to the loss of target feature information.

**Methods:**

In this paper, we propose a non-convex sparse regularization model that employs the Cauchy function. By designing a proximal gradient algorithm, our proposed model achieves closer-to-unbiased estimation than existing sparse models. Therefore, it can learn more accurate, discriminative, and effective feature information. Additionally, the proposed method can perform feature selection and classification simultaneously, without requiring additional classifiers.

**Results:**

We conducted experiments on two publicly available motor imagery EEG datasets. The proposed method achieved an average classification accuracy of 82.98% and 64.45% in subject-dependent and subject-independent decoding assessment methods, respectively.

**Conclusion:**

The experimental results show that the proposed method can significantly improve the performance of motor imagery decoding, with better classification performance than existing feature selection and deep learning methods. Furthermore, the proposed model shows better generalization capability, with parameter consistency over different datasets and robust classification across different training sample sizes. Compared with existing sparse regularization methods, the proposed method converges faster, and with shorter model training time.

## 1. Introduction

Motor imagery-based brain-computer interface (BCI) systems have been widely applied in stroke rehabilitation, neuroprosthetics, and robot control (Liao et al., 2023). However, motor imagery electroencephalogram (EEG) signals are spontaneous, with poor signal quality and large individual differences, resulting in low accuracy and poor stability of motor imagery decoding (Zhang et al., 2021). Currently, motor imagery decoding is still a big challenge.

The time, frequency, and space information of EEG signals is crucial for motor imagery decoding (Zheng et al., 2022). Therefore, temporal-frequency-spatial feature-based motor imagery decoding has been widely studied (Chen et al., 2023). In the process of temporal-frequency-spatial feature extraction, the original EEG signals are first decomposed into multiple time-frequency units, then the common spatial pattern (CSP) algorithm is used to extract the spatial features on each time-frequency unit, and finally, the spatial features of multiple time-frequency units are cascaded into a feature vector (Miao et al., 2021), which significantly increases the feature dimension of EEG. The number of feature dimensions exceeds one hundred or even several hundred, while feature redundancy exists. In addition, due to the difficulty and high cost of collecting EEG samples, especially for patients, the sample size is generally relatively small, usually only a few dozen. Therefore, the temporal-frequency-spatial feature is high-dimensional small-sample data, which will bring a series of problems to the EEG classification model, such as the problem of overfitting and model solution underdetermination (Chadebec et al., 2022).

For high-dimensional small-sample problems, feature selection is an effective method (Chen et al., 2023), which can remove redundant information, reduce the feature dimension, simplify the model complexity, and effectively solve many problems (Shen and Zhang, 2022). Sparse regularization-based feature selection methods are commonly used in motor imagery decoding, such as the least absolute shrinkage and selection operator (LASSO; Zhang et al., 2022), group LASSO (gLASSO; Zhang et al., 2020), and sparse group LASSO (sgLASSO; Jiao et al., 2018). These methods are all convex sparse regularization models, which penalize the regression coefficients of the model by the $l_{1}$ norm so that regression coefficients with small absolute values are automatically compressed to zero, thus generating sparse solutions and achieving feature selection. However, the $l_{1}$ norm is a biased estimation that penalizes all components of the regression coefficients to the same extent. In addition to compressing the regression coefficients corresponding to the noisy features to zero, a certain degree of compression is applied to the target features, resulting in a biased estimation of the target features. Therefore, the biased estimation model applied to feature selection will result in the loss of useful information and degrade the classification performance.

Non-convex sparse regularization models penalize the regression coefficients to different degrees for different values of the regression coefficients, which are approximate unbiased estimation models and have stronger noise suppression and sparsity induction capabilities (Wang et al., 2018). The commonly used non-convex regularization models, such as smoothly clipped absolute deviation (SCAD; Chopra and Lian, 2010) and minimax concave penalty (MCP; You et al., 2019) models, have been widely used in the fields of image restoration and image denoising, and their effect is remarkable. SCAD and MCP models penalize the regression coefficients in chunks, reducing the compression of the regression coefficients corresponding to the target features and alleviating the biased estimation problem of the $l_{1}$ norm to some extent (Wen et al., 2018). However, the SCAD and MCP models may still compress the regression coefficients of a portion of the target features. Therefore, there is still a need to explore new non-convex sparse regularization methods to better address the biased estimation problem and learn more accurate, discriminative, and effective feature information.

In addition, many deep learning methods for temporal-frequency-spatial feature learning have been proposed, which are mostly inspired by the FBCSP approach (Ang et al., 2008) in a machine learning framework, using convolutional neural network (CNN) for frequency band filtering followed by spatial filtering (Zancanaro et al., 2021). Earlier and more classical works include ConvNets (Schirrmeister et al., 2017) and EEGNet (Lawhern et al., 2018). There are also works that use traditional band-pass filtering banks to decompose the raw EEG signal into multiple frequency subbands and then use CNN to learn spatial domain or time domain information, such as FBCNet (Mane et al., 2020), FBMSNet (Liu et al., 2022), and the literature (Sakhavi et al., 2018; Kwon et al., 2019; Dai et al., 2020). Subsequent work uses multiscale convolution to learn frequency domain information in parallel and then learns either spatial domain or time domain information at each branch, such as MSFBCNN (Wu et al., 2019), MMCNN (Jia et al., 2020), and the literature (Chang et al., 2022; Li et al., 2023). Deep learning methods have a strong representation learning capability but require a large number of data samples (Autthasan et al., 2021). Although deep learning has been widely used in motor imagery decoding, feature selection is integrated into the overall network structure, and the theoretical support and physiological interpretability are relatively poor. Furthermore, the model training is time-consuming.

A new non-convex sparse regularization model is proposed to deal with high-dimensional small-sample problems for motor imagery decoding in this paper, which can learn more accurate, discriminative, and effective temporal-frequency-spatial features. Specifically, we propose a non-convex sparse regularization model based on the Cauchy function and design an effective solution algorithm based on the proximal gradient. The proposed model penalizes the weight coefficients of each feature independently with a better ability to induce sparsity while avoiding the compression of the weight coefficients of the target features to zero during noise suppression, which achieves approximately unbiased estimation. We conducted experiments on two publicly available motor imagery EEG datasets to fully and adequately validate the effectiveness of the proposed model, using subject-dependent and subject-independent assessment methods.

The main contributions and innovations of this paper are summarized below.

1. A new non-convex sparse regularization model based on the Cauchy function is proposed, which penalizes the weight coefficients of each feature independently, avoiding the compression of the weight coefficients of the target features to zero during noise suppression, thereby achieving closer-to-unbiased estimation than existing sparse models. Therefore, it can learn more accurate, discriminative, and effective feature information.
2. In addition to the proposed non-convex sparse regularization model, we introduced two other existing non-convex models (SCAD and MCP) for EEG temporal-frequency-spatial feature learning and demonstrated the effectiveness of the non-convex sparse regularization model in EEG decoding. The non-convex sparse regularization model significantly outperformed the convex sparse regularization model in subject-dependent decoding.
3. We conducted a comprehensive and adequate validation of the effectiveness of the proposed methods using subject-dependent and subject-independent assessment methods. Comparisons are made with nine existing feature selection methods and four deep learning methods. For filtered and wrapped methods, five classifiers are combined for data experiments.

The rest of this paper is organized as follows. Section II presents the experimental data; Section III introduces the temporal-frequency-spatial feature extraction method, the EEG decoding framework, and the Cauchy non-convex sparse regularization model proposed in this paper; Section IV presents and analyzes the experimental results. Sections V and VI are discussion and conclusion, respectively.

## 2. Data description

The proposed method was validated using two publicly available motor imagery EEG datasets, Dataset 1 from the international BCI competition and Dataset 2 from the database of the BNCI Horizon 2020 project with no. 002–2014. The basic information of both datasets is shown in Table 1, other detailed information can be found on the official website.

**Table 1:** Description of all datasets.

Datasets	Number of channels	Sampling rate	Number of subjects	Tasks	Number of training and test sets for each subject	Data access website
Dataset 1 (Liu et al., 2022)	22	250 Hz	9	Left-hand, right-hand, foot, tongue	288, 288	https://www.bbci.de/competition/iv/
Dataset 2 (Autthasan et al., 2021)	15	512 Hz	14	Right-hand, foot	100, 60	http://bnci-horizon-2020.eu/database/data-sets

For Dataset 1, we only study the binary classification problems, so the four types of tasks are arranged and combined to obtain six sets of binary classification problems (Zhang et al., 2022), namely, L vs. R, L vs. F, L vs. T, R vs. F, R vs. T, and F vs. T, where L vs. R denotes the left-hand and right-hand motor imagery tasks, the rest can be deduced accordingly. Note that, for the binary classification task, the number of both training and test sets is 144. For Dataset 2, the original data are downsampled to 256 Hz in this paper.

## 3. Methods

This section first introduces the temporal-frequency-spatial feature extraction method, then describes the EEG decoding framework based on temporal-frequency-spatial features, and finally proposes the non-convex sparse regularization model based on the Cauchy function.

### 3.1. Temporal-frequency-spatial feature extraction

**Figure 1:**
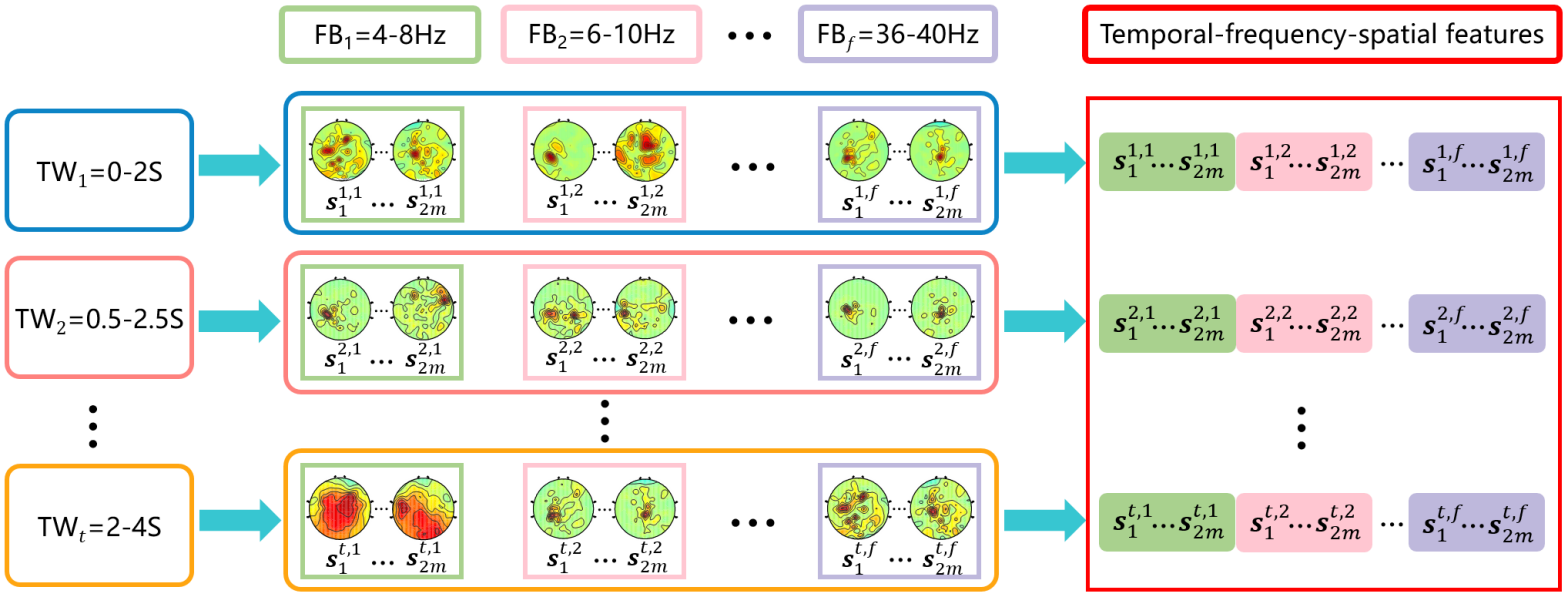
Temporal-frequency-spatial feature extraction. Time window interception is performed first, followed by frequency band filtering, and finally CSP features are extracted on each time-frequency unit.

As shown in Figure 1, the temporal-frequency-spatial feature extraction mainly consists of three steps. First, time window segmentation. A sliding time window is used to intercept the original EEG signal to obtain 5 time windows with a length of 2 s and an overlap rate of 0.5 s, i.e., 0–2 s, 0.5–2.5 s, …, 2–4 s. Second, band-pass filtering. Each time window is filtered with a band-pass filter bank to obtain 17 sub-bands with a width of 4 Hz and an overlap rate of 2 Hz, i.e., 4–8 Hz, 6–10 Hz, …, 36–40 Hz, the 6th-order Butterworth filter is selected. After the above signal processing, the original EEG signal is divided into a total of 85 time-frequency units. Third, feature extraction. For each time-frequency unit, the CSP method is used to extract the spatial features separately, thus obtaining multiple groups of temporal-frequency-spatial features containing rich EEG information. In this paper, the pair number of the spatial filter for CSP is set to be 1 (Blankertz et al., 2008; Lotte and Guan, 2011), i.e., each time-frequency unit contains two spatial features.

### 3.2. EEG decoding framework

**Figure 2:**
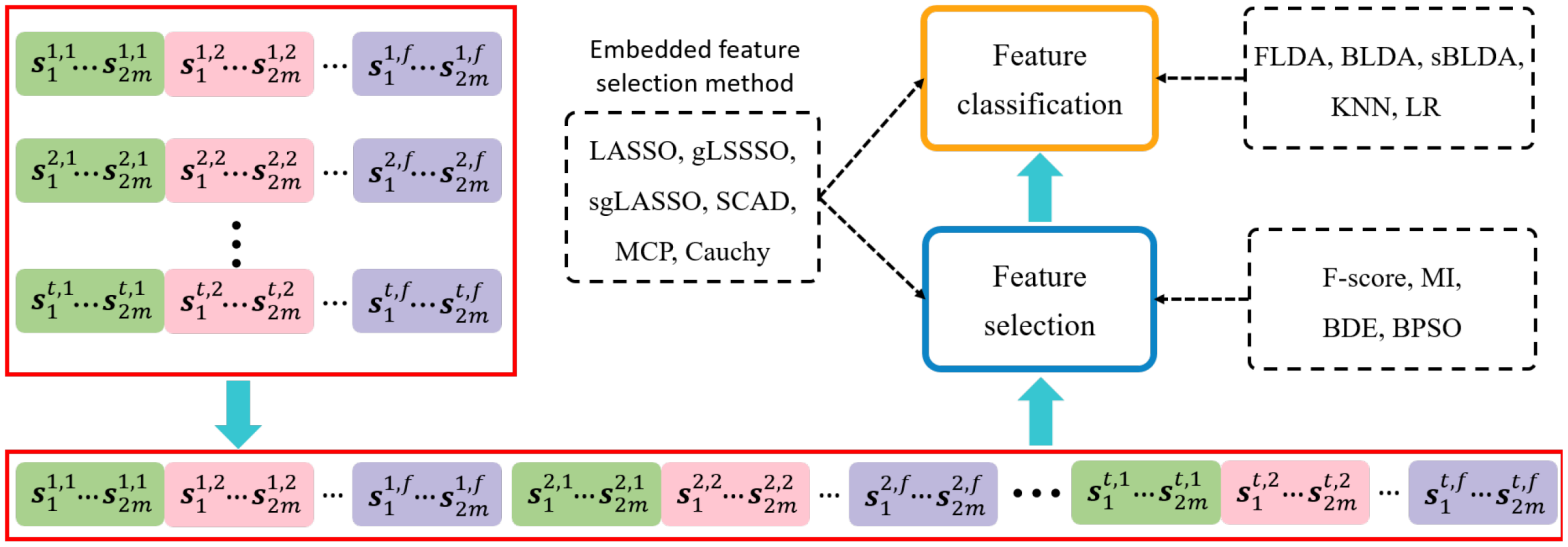
EEG decoding framework. The dashed boxes indicate the specific implementation methods of each data processing step. The embedded feature selection method based on sparse regularization performs feature selection and classification simultaneously.

The EEG decoding framework is shown in Figure 2, where each group of temporal-frequency-spatial features is cascaded by the time window and frequency band to obtain a feature vector. One motor imagery task corresponds to one feature vector, and feature vectors from multiple tasks will form a sample matrix, each row of which is a sample and each column is a one-dimensional feature. Feature selection and classification are performed sequentially on the sample matrix. Filtered and wrapped methods need to be configured with additional classifiers, and the embedded methods can perform feature selection and classification simultaneously.

The contents of the dashed boxes indicate the specific algorithms used for feature selection and feature classification, respectively. The feature selection methods used in this paper include Fisher score (F-score; Radman et al., 2021), mutual information (MI; Park et al., 2018), binary differential evolution (BDE; Datta and Dutta, 2012), binary particle swarm optimization (BPSO; Too et al., 2019), LASSO (Zhang et al., 2022), gLASSO (Zhang et al., 2020), sgLASSO (Jiao et al., 2018), SCAD (Chopra and Lian, 2010), MCP (You et al., 2019), and Cauchy. The classifiers configured for filtered and wrapped methods include Fisher linear discriminant analysis (FLDA; Hoffmann et al., 2008), Bayesian linear discriminant analysis (BLDA; Hoffmann et al., 2008), sparse BLDA (sBLDA; Bishop and Nasrabadi, 2006; Hoffmann et al., 2008), K-Nearest Neighbor (KNN), and Logistic Regression (LR).

Cauchy is a newly proposed embedded feature selection method. Next, we will introduce the Cauchy non-convex sparse regularization model in detail.

### 3.3. Cauchy non-convex sparse regularization model

The general mathematical model of the embedded feature selection method based on sparse regularization is as follows:


(1) $$\min_{w}\frac{1}{2}∥y-Xw{∥}_{2}^{2}+\lambda P\left(w\right)$$

where $X={\left(x_{1},x_{2},\cdots ,x_{N}\right)}^{T}\in R^{N\times P}$ denotes the sample matrix, N is the total number of samples, and P is the feature dimension of one sample. $w={\left(w_{1},w_{2},\cdots ,w_{P}\right)}^{T}\in R^{P}$ is the model regression coefficient vector, which represents the weight magnitude of the features. $y={\left(y_{1},y_{2},\cdots ,y_{N}\right)}^{T}\in R^{N}$ denotes the sample labels, and $y_{i}\in \left\{-1,1\right\}$. $∥•{∥}_{2}^{2}$ denotes the square of the $l_{2}$ norm, and λ>0 denotes the regularization parameter. The first term of Eq. (1) is the data fidelity term and the second term is the penalty term. $P\left(w\right)$ is a function of the coefficient vector w, which penalizes or constraints w. During model training, some regression coefficients in w are compressed to zero by $P\left(w\right)$. The features corresponding to a coefficient of zero will not work in the model fit. Therefore, sparse regularization models can simultaneously achieve feature selection and classification. When $P\left(w\right)$ takes a different penalty function, the model will obtain solutions with different structures.

LASSO is a biased convex sparse model, and its specific mathematical model is as follows (Zhang et al., 2022).


(2) $$\underset{w}{w\;=\;\arg\min}\frac{1}{2}∥y-Xw{∥}_{2}^{2}+\lambda ∥w{∥}_{1}$$

where $P\left(w\right)=\;∥w{∥}_{1}=\sum_{i=1}^{P}\left|w_{i}\right|$, $∥•{∥}_{1}$ denotes the $l_{1}$ norm, $\left|w_{i}\right|$ denotes the absolute value of $w_{i}$. The LASSO model penalizes all components of the regression coefficients to the same extent, which results in a biased estimation. Similarly, gLASSO (Zhang et al., 2020) and sgLASSO (Jiao et al., 2018) are also biased convex sparse models.

#### 3.3.1. Existing non-convex sparse regularization models

Non-convex sparse regularization are approximate unbiased estimation models, which have stronger noise suppression and sparsity induction capabilities than convex sparse regularization (Wang et al., 2018). In the following, we will provide a detailed introduction to two existing non-convex sparse regularization methods, namely SCAD and MCP.

SCAD is an approximate unbiased non-convex sparse model with the following objective function (Chopra and Lian, 2010).


(3) $$\underset{w}{w=\arg\min}\frac{1}{2}∥y-Xw{∥}_{2}^{2}+\sum_{p=1}^{P}\varphi_{{\;}_{\lambda ,\gamma }}^{1}\left(w_{p}\right)$$

where $p\in \left\{1,\cdots ,P\right\}$, $\varphi_{\lambda ,\gamma }^{1}\left(•\right)$ are SCAD penalties, defined as


(4) $$\varphi_{{\;}_{\lambda ,\gamma }}^{1}\left(\theta \right)=\left\{ \begin{array}{l} \lambda \left|\theta \right|,0\leq \left|\theta \right|\leq \lambda \\ -\frac{\left({\left|\theta \right|}^{2}-2\gamma \lambda \left|\theta \right|+\lambda^{2}\right)}{2\left(\gamma -1\right)},\lambda <\left|\theta \right|<\gamma \lambda \\ \frac{\left(\gamma +1\right)\lambda^{2}}{2},\left|\theta \right|\geq \gamma \lambda \end{array}\right.$$

where λ>0 is the regularization parameter. γ>2, γ is set to 3 in this paper. SCAD compresses each weight coefficient to different degrees. The compression of the weight coefficients corresponding to the noise variables (absolute values less than λ) has the same effect as the LASSO model, which tends to compress this part of the weight coefficients to zero; the compression of the weight coefficients corresponding to the target variables is gradually reduced. Since SCAD reduces or even avoids the compression of the weight coefficients corresponding to the target variables, it effectively overcomes the biased estimation of LASSO and improves its parameter estimation consistency and variable selection consistency.

MCP is also an approximate unbiased non-convex sparse model with the following objective function (You et al., 2019).


(5) $$\underset{w}{w=\arg\min}\frac{1}{2}∥y-Xw{∥}_{2}^{2}+\sum_{p=1}^{P}\varphi_{\lambda ,\gamma }^{2}\left(w_{p}\right)$$

where $p\in \left\{1,\cdots ,P\right\}$, $\varphi_{\lambda ,\gamma }^{2}\left(•\right)$ are MCP penalties, defined as


(6) $$\varphi_{\lambda ,\gamma }^{2}\left(\theta \right)=\left\{ \begin{array}{l} \lambda \left|\theta \right|-\frac{{\left|\theta \right|}^{2}}{2\gamma },\;\left|\theta \right|\leq \gamma \lambda \\ \frac{1}{2}\gamma \lambda^{2},\;\left|\theta \right|>\gamma \lambda \end{array}\right.$$

where γ>1, γ is set to 2 in this paper. Similar to SCAD, MCP also compresses each weight coefficient to different degrees. MCP compresses the weight coefficients corresponding to the noise variables (absolute values less than γλ), while it does not compress the weight coefficients corresponding to the target variables (absolute values greater than γλ). Thus, MCP also achieves approximately unbiased estimation.

The SCAD and MCP models penalize the regression coefficients in chunks, which mitigates the biased estimation problem to some extent, but still inaccurately compresses a portion of the target features.

#### 3.3.2. The proposed Cauchy regularization models

To better solve the biased estimation problem in temporal-frequency-spatial feature selection, we propose a non-convex sparse regularization model based on the Cauchy function. The Cauchy function is defined as follows:


(7) $$\varphi_{C}\left(x\right)=-\log\left(\frac{\gamma }{\gamma^{2}+x^{2}}\right)$$

where γ≥0. In this paper, the Cauchy function is used as a penalty term $P\left(w\right)$, and a new non-convex regularized feature selection model is constructed, the mathematical model of which is specified as follows:


(8) $$w=\underset{w}{\arg\min}\frac{1}{2}∥y-Xw{∥}_{2}^{2}-\lambda \log\left(\frac{\gamma }{\gamma^{2}+∥w{∥}_{1}^{2}}\right)$$

where $∥•{∥}_{1}^{2}$ denotes the square of the $l_{1}$ norm. This concave log function imposes an uneven penalty on all regression coefficients (Zhang et al., 2020). It allows a larger penalty to be imposed on small-valued elements than on larger-valued elements, a property that makes the log function closer to unbiased estimation than the SCAD and MCP models. Also, the Cauchy model has a better ability to induce sparsity than the $l_{1}$ norm (Zhang et al., 2020).

In this paper, the Cauchy non-convex regularized model is solved in two parts and iterated cyclically until convergence. The specific procedure is as follows:

1. Gradient solution. Gradient solution of the differentiable term of the model with an intermediate point $v_{\tau }$ in the τ step iteration:

(9) $$v_{\tau }=w_{\tau }-1/\beta \left(X^{T}\left(Xw_{\tau }-y\right)\right)$$

where $\beta =\;∥X^{T}X{∥}_{2}$, $w_{\tau }$ denote the feature weights of the τ step iteration.

(2) Proximity operator solution. Compute the proximity operator of the Cauchy function at the intermediate point $v_{\tau },$ i.e.


(10) $$\begin{array}{l} w_{\tau +1}={\text{prox}}_{\beta ,\text{Cauchy}}\left(v_{\tau }\right)\\ \;={\text{prox}}_{\beta ,\text{Cauchy}}\left(w_{\tau }-1/\beta \left(X^{T}\left(Xw_{\tau }-y\right)\right)\right) \end{array}$$

where ${\text{prox}}_{\beta ,\text{Cauchy}}\left(v_{\tau }\right)$ is the proximity operator of the Cauchy function, defined as follows (Karakuş et al., 2020):


(11) $${\text{prox}}_{\beta ,\text{Cauchy}}\left(v_{\tau }\right)=\underset{w}{\arg\min}\left\{\frac{\beta }{2}∥w-v_{\tau }{∥}_{2}^{2}-\lambda \log\left(\frac{\gamma }{\gamma^{2}+∥w{∥}_{1}^{2}}\right)\right\}$$

Find the partial derivative of Eq. (9) with respect to w and make it zero, i.e.


(12) $${w_{\tau +1}}^{3}-v_{\tau }w_{\tau +1}^{2}+\left(\gamma^{2}+\frac{2\lambda }{\beta }\right)w-v_{\tau }\gamma^{2}=0$$

Next, the Cardano method (Karakuş et al., 2020) is used to solve for $w_{\tau +1}$ in Eq. (12), i.e.


(13) $$\begin{array}{l} w_{\tau +1}=\frac{v_{\tau }}{3}+\sqrt[{3}]{q/2+\sqrt{p^{3}/27+q^{2}/4}}\\ \;+\sqrt[{3}]{q/2-\sqrt{p^{3}/27+q^{2}/4}} \end{array}$$

where $p=\gamma^{2}+\frac{2\lambda }{\beta }-\frac{v_{\tau }^{2}}{3}$,$q=\frac{2v_{\tau }^{3}}{27}+v_{\tau }\gamma^{2}-\frac{v_{\tau }}{3}\left(\gamma^{2}+\frac{2\lambda }{\beta }\right)$. In this paper, γ is set to 0.007 in subject-dependent decoding and 0.003 in subject-independent decoding.

## 4. Experiments

### 4.1. Evaluation indicators and assessment methods

For each subject, the classification accuracy of the test set is used as an evaluation indicator, i.e., the number of correctly classified samples divided by the total number of test set samples. Two assessment methods, subject-dependent and subject-independent decoding, are used to verify the classification performance of the proposed method. For subject-dependent decoding, one model is trained for each subject, and the division of the training and test sets of the model is kept consistent with the original data set, as detailed in the data description section.

For subject-independent decoding, the training and test sets of all subjects except the target subject are used to train the model, and the test set of the target subject is used to evaluate the performance of the model. For example, if subject 1 in Dataset 1 is selected as the target subject, all training and test sets of the other 8 subjects constitute the training set of the model, and the test set of subject 1 constitutes the test set of the model.

### 4.2. Comparison methods and model parameter settings

There are nine feature selection methods involved in the comparison. F-score and MI are filtered methods, and BDE and BPSO are wrapped methods. LASSO, gLASSO, sgLASSO, SCAD, MCP, and Cauchy are embedded methods, among which LASSO, gLASSO, and sgLASSO are based on convex sparse regularization, and SCAD, MCP, and Cauchy are based on non-convex sparse regularization. We further divide the training set of the model into a training subset and a validation set and use the average accuracy of 10 cross-validations as the selection criterion for the optimal model.

The F-score and MI methods first rank the features using their respective metric criteria and finally select the optimal feature subset using 10-fold cross-validation and the classifier. After the optimal feature subset is obtained by the BDE and BPSO methods, it is directly fed into the classifier for classification. The model parameters of the BDE and BPSO methods are set following the literature (Datta and Dutta, 2012; Too et al., 2019). The alternative sets of regularization parameters for the LASSO, gLASSO, sgLASSO, SCAD, MCP, and Cauchy methods are set as $\left\{2^{-5},2^{-4.8},\cdots ,2^{4.8},2^{5}\right\}$, and the optimal regularization parameters are selected using 10-fold cross-validation. γ is set to 3 and 2 in the SCAD and MCP models, respectively.

There are five classifiers used for filtered and wrapped methods, including FLDA, BLDA, sBLDA, KNN, and LR. The K value of the KNN classifier is set to 5, and no parameters need to be set for other classifiers.

### 4.3. Experimental results

#### 4.3.1. Subject-dependent decoding

The classification results of all feature selection methods in subject-dependent decoding are listed in Table 2. Due to the limited space, only the average classification accuracy is listed for each dataset, which is obtained by averaging the classification accuracies of all subjects within the dataset. The classification results for Dataset 1 were obtained by averaging the classification accuracies of all subjects in the six sets of binary classification tasks. From Table 2 we can see that the proposed Cauchy feature selection method achieves the highest classification accuracy on both Dataset 1 and Dataset 2.

**Table 2:** Classification accuracy of various feature selection methods in subject-dependent decoding.

Methods		Dataset 1 (L vs. R)	Dataset 1 (L vs. F)	Dataset 1 (L vs. T)	Dataset 1 (R vs. F)	Dataset 1 (R vs. T)	Dataset 1 (F vs. T)	Dataset 1	Dataset 2
Classifier	Feature Selection								
FLDA	F-score	78.55	86.04	83.41	83.80	77.70	77.16	81.11	70.83
	MI	75.31	82.72	82.72	82.64	79.78	74.30	79.58	70.36
	BDE	73.07	81.56	79.32	78.78	77.08	69.45	76.54	63.93
	BPSO	71.68	83.64	78.70	77.55	76.47	71.06	76.52	65.00
BLDA	F-score	80.32	86.26	82.48	86.27	82.72	78.78	82.81	76.07
	MI	80.71	85.80	81.71	**86.96**	83.87	77.78	82.81	73.09
	BDE	79.78	86.73	82.64	84.34	82.95	74.23	81.78	72.74
	BPSO	78.63	87.35	81.25	83.41	82.56	75.77	81.49	72.26
sBLDA	F-score	78.16	85.49	84.95	86.03	81.40	75.23	81.88	76.19
	MI	77.93	85.80	82.41	84.26	80.71	76.31	81.24	74.17
	BDE	75.39	84.64	82.02	84.57	80.17	72.07	79.81	68.81
	BPSO	76.39	85.80	81.17	81.17	81.48	73.69	79.95	71.19
KNN	F-score	80.71	84.95	85.03	84.88	83.64	80.17	83.23	77.98
	MI	82.25	85.80	83.72	86.19	82.02	79.47	83.24	77.62
	BDE	78.09	86.34	83.18	81.40	82.48	77.93	81.57	72.74
	BPSO	78.32	85.88	84.03	82.10	82.25	77.55	81.69	71.67
LR	F-score	80.40	85.88	84.18	85.34	82.41	79.63	82.97	76.55
	MI	79.78	82.10	82.64	84.57	81.87	78.16	81.52	73.69
	BDE	70.37	78.09	78.01	75.46	74.07	67.59	73.93	56.90
	BPSO	70.14	80.63	78.24	74.31	74.85	69.44	74.60	59.29
LASSO		77.32	86.65	81.87	84.49	81.33	75.31	81.16	73.10
gLASSO		77.55	85.80	80.63	82.41	83.41	77.08	81.15	72.74
sgLASSO		72.14	81.17	80.25	80.55	77.78	73.69	77.60	73.69
SCAD		81.09	87.65	**86.34**	84.34	83.49	78.78	83.62	75.95
MCP		81.09	87.65	86.27	84.26	83.49	78.47	83.54	75.95
Cauchy		**82.95**	**89.05**	83.34	84.03	**84.11**	**81.10**	**84.09**	**78.69**

Dataset 1 (L vs. R) denotes a subset of Dataset 1, i.e., the subset of data corresponding to the left-hand and right-hand motor imagery tasks, the others can be deduced accordingly. Bold display indicates that the method is optimal.

**Figure 3:**
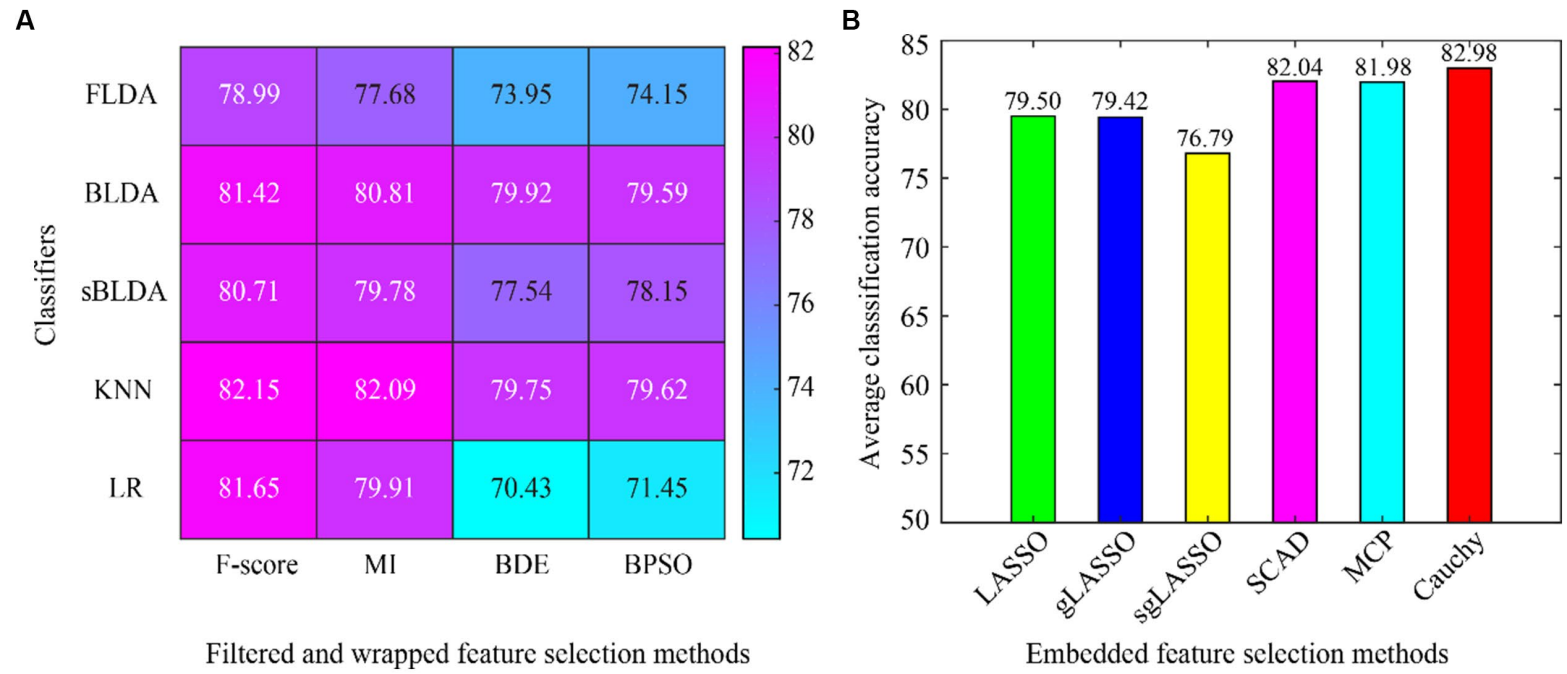
Average classification accuracy of all data in subject-dependent decoding. **(A)** Filtered and wrapped methods combined with 5 classifiers. **(B)** Embedded methods perform feature selection and classification simultaneously, without additional classifiers.

The average classification accuracy for all data is shown in Figure 3, which is obtained by averaging the classification accuracies of all subjects in Dataset 1 and Dataset 2. From the overall results of Figure 3, the existing embedded methods (LASSO, gLASSO, sgLASSO, SCAD, and MCP) have little or no advantage over the filtered and wrapped methods. However, the proposed method in this paper has a clear advantage. In addition, the non-convex regularization method outperforms the convex regularization method, which proves the superiority of the non-convex regularization method.

#### 4.3.2. Subject-independent decoding

The classification results of all feature selection methods in subject-independent decoding are listed in Table 3. Similar to Table 2, only the average classification accuracy for each dataset is listed. The proposed Cauchy method achieves the best classification results on Dataset 1. Although Cauchy is not optimal on Dataset 2, it is not far from the highest value and outperforms the vast majority of existing methods.

**Table 3:** Classification accuracy of various feature selection methods in subject-independent decoding.

Methods		Dataset 1 (Lvs R)	Dataset 1 (L vs. F)	Dataset 1 (L vs. T)	Dataset 1 (R vs. F)	Dataset 1 (R vs. T)	Dataset 1 (F vs. T)	Dataset 1	Dataset 2
Classifier	Feature Selection								
FLDA	F-score	66.59	62.50	61.57	62.50	62.58	58.87	62.44	63.33
	MI	66.67	61.57	62.73	62.96	61.65	58.49	62.35	63.69
	BDE	67.82	62.27	62.58	62.96	62.11	59.03	62.80	66.31
	BPSO	68.06	62.11	62.27	63.66	61.88	58.03	62.67	63.69
BLDA	F-score	68.05	63.27	63.20	63.50	63.12	58.57	63.29	64.05
	MI	68.06	63.27	62.34	**63.81**	63.12	57.72	63.05	65.36
	BDE	**68.75**	63.19	62.96	63.35	63.66	59.26	63.53	65.72
	BPSO	68.44	62.81	62.27	62.42	62.65	58.72	62.89	64.29
sBLDA	F-score	68.75	61.65	64.04	62.50	63.04	56.40	62.73	65.72
	MI	66.13	62.35	**64.58**	62.89	62.04	57.41	62.56	**66.55**
	BDE	68.52	62.65	63.58	63.35	63.58	57.87	63.26	65.24
	BPSO	67.28	62.66	62.58	63.12	61.65	57.10	62.40	64.17
KNN	F-score	60.80	58.95	58.95	57.41	58.18	55.40	58.28	57.62
	MI	62.27	59.33	58.10	56.79	59.18	54.71	58.40	55.83
	BDE	60.96	58.03	57.64	57.95	58.49	54.40	57.91	59.29
	BPSO	62.19	59.18	58.64	58.03	58.10	56.56	58.78	56.55
LR	F-score	68.21	62.81	62.19	62.27	61.73	58.03	62.54	64.40
	MI	66.74	61.42	63.35	63.27	61.11	57.33	62.20	64.05
	BDE	67.67	61.81	62.73	62.89	61.57	59.10	62.63	65.72
	BPSO	68.06	61.26	62.35	63.58	61.65	58.03	62.49	63.93
LASSO		67.36	64.66	63.12	62.73	**66.13**	59.65	63.94	64.41
gLASSO		66.20	66.28	60.65	61.03	63.89	58.80	62.81	62.50
sgLASSO		66.74	68.37	60.19	61.42	61.50	59.34	62.92	65.24
SCAD		63.19	64.35	60.57	62.73	64.20	58.49	62.26	62.26
MCP		63.35	64.43	60.57	62.65	63.74	58.10	62.14	62.62
Cauchy		66.59	**68.83**	62.11	62.19	64.20	**60.80**	**64.12**	65.71

Dataset 1 (L vs. R) denotes a subset of Dataset 1, i.e., the subset of data corresponding to the left-hand and right-hand motor imagery tasks, the others can be deduced accordingly. Bold display indicates that the method is optimal.

**Figure 4:**
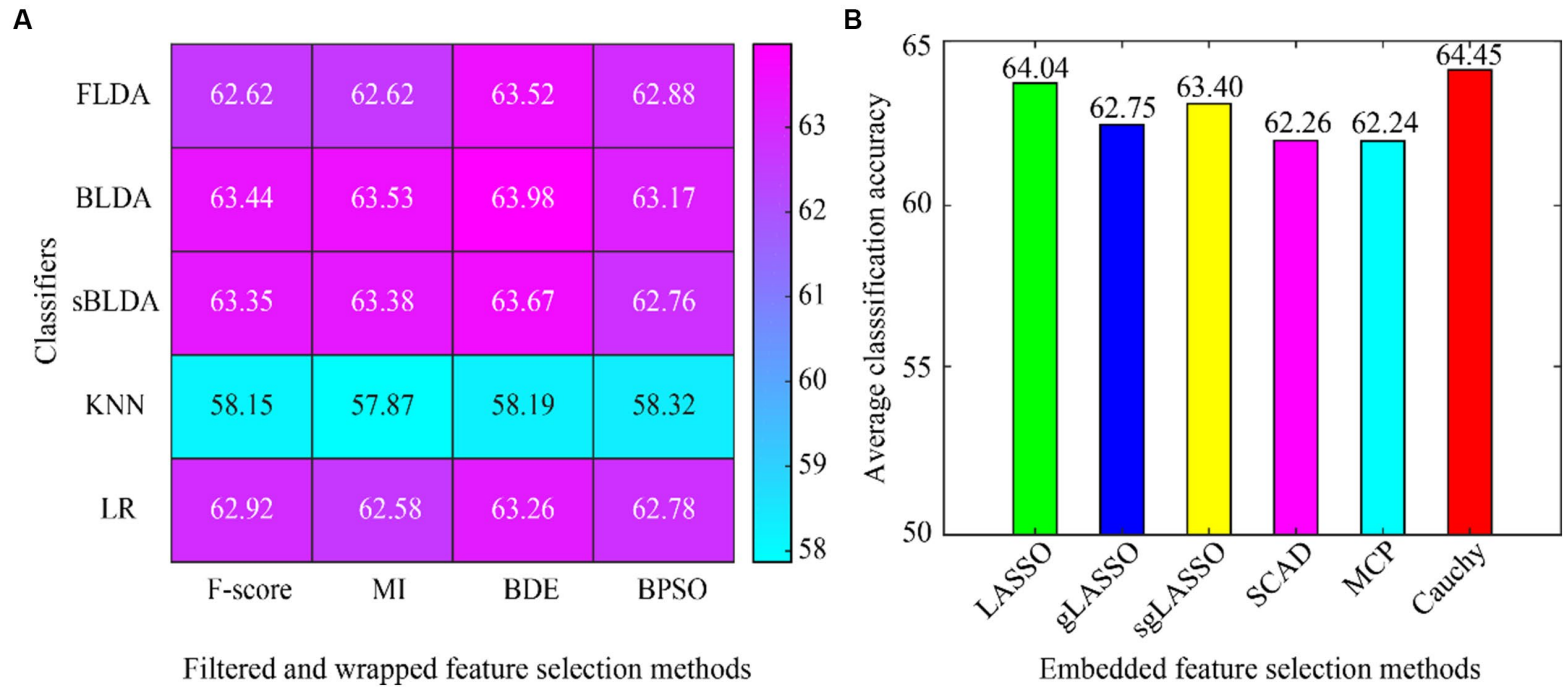
Average classification accuracy of all data in subject-independent decoding. **(A)** Filtered and wrapped methods combined with 5 classifiers. **(B)** Embedded methods perform feature selection and classification simultaneously, without additional classifiers.

The average classification accuracy for all the data is shown in Figure 4, which is obtained by averaging the classification accuracies of all subjects in Dataset 1 and Dataset 2. As can be seen from Figure 4, the Cauchy method still achieves the best classification results, followed closely by the LASSO method. In subject-independent decoding, the existing convex regularization methods (LASSO, gLASSO, sgLASSO) outperformed the non-convex regularization methods (SCAD and MCP). The filtered and wrapped methods performed comparably or even better than the existing embedded methods. However, the results of filtered and wrapped methods based on KNN classifiers are very poor. This indicates that some classifiers are suitable for subject-dependent decoding but not for subject-independent decoding.

#### 4.3.3. Compared with deep learning methods

In sections 4.3.1 and 4.3.2, the compared methods belong to machine learning methods. In this section, the proposed method is compared with deep learning methods. Deep ConvNet (Schirrmeister et al., 2017), EEGNet-8,2 (Lawhern et al., 2018), Spectral-Spatial CNN (Kwon et al., 2019), and MIN2NET (Autthasan et al., 2021) perform temporal-frequency-spatial feature learning for EEG decoding in different ways. In Table 4, we directly cite the experimental results provided in the literature (Autthasan et al., 2021) without reproducing these deep learning methods. From Table 4 we can see that the proposed method has significant advantages in subject-dependent decoding. In subject-independent decoding, the proposed method is optimal on Dataset 1 (L vs. R) and second only to Spectral-Spatial CNN on Dataset 2 (Kwon et al., 2019).

**Table 4:** Classification accuracy of the proposed method and deep learning methods.

Assessment methods	Datasets	Deep ConvNet (Schirrmeister et al., 2017)	EEGNet-8,2 (Lawhern et al., 2018)	Spectral-Spatial CNN (Kwon et al., 2019)	MIN2NET (Autthasan et al., 2021)	Cauchy
Subject-dependent	Dataset 1 (L vs. R)	63.72 ± 17.18	65.93 ± 18.44	76.91 ± 13.75	65.23 ± 16.14	**82.95 ± 12.14**
	Dataset 2	61.40 ± 15.66	67.76 ± 18.09	76.76 ± 16.66	65.90 ± 16.50	**78.69 ± 14.79**
Subject-independent	Dataset 1 (L vs. R)	56.34 ± 8.86	64.26 ± 11.03	66.05 ± 13.70	60.03 ± 9.24	**66.59** ± 12.86
	Dataset 2	65.26 ± 16.83	58.07 ± 11.45	**66.21** ± 15.15	59.79 ± 13.72	65.71 ± 16.97

Bold values indicate that the method achieved the best classification results on a particular dataset.

#### 4.3.4. Model generalization ability of the Cauchy method

The model generalization ability of the proposed Cauchy method is analyzed from two aspects. First, the parameter consistency over different datasets. Second, the classification performance across different training sample sizes.

**Figure 5:**
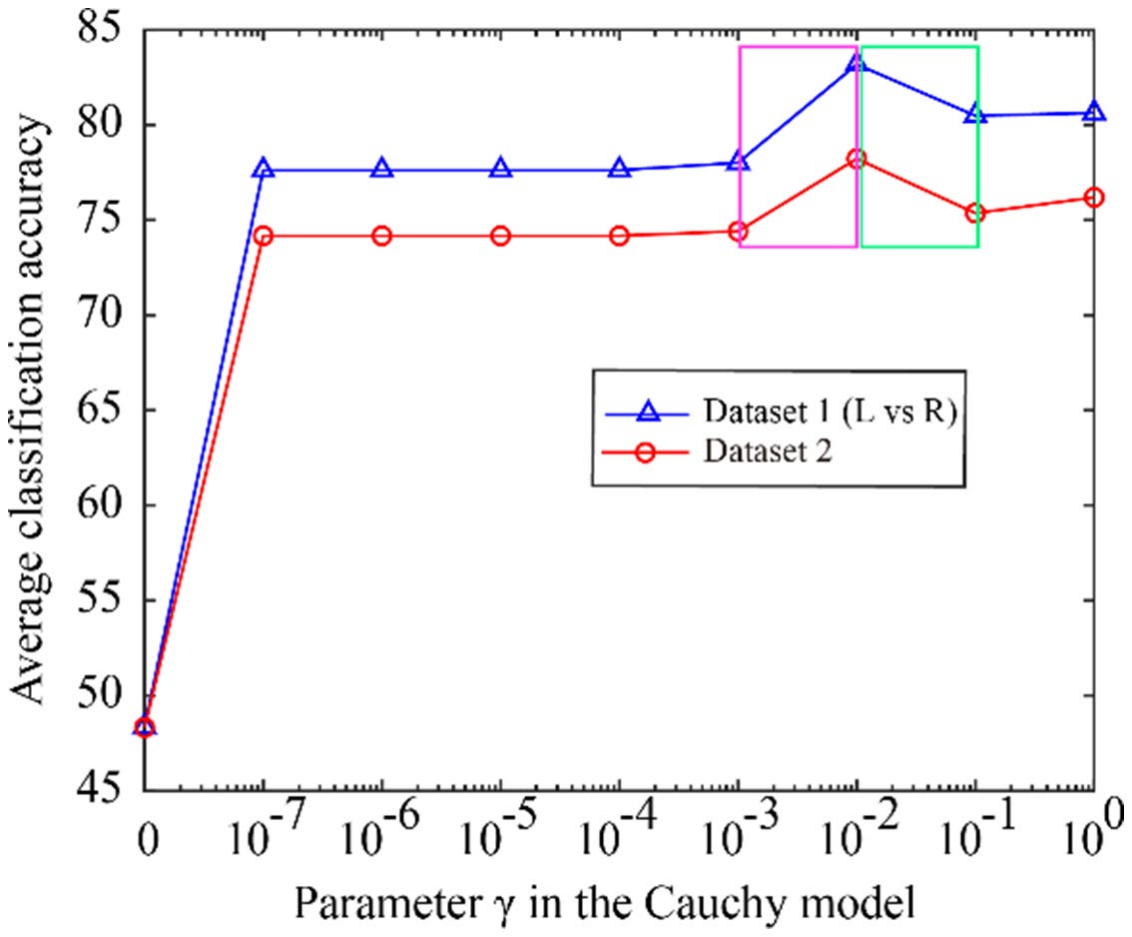
The classification accuracy varies with the Cauchy’s. model parameter γ, ranging from 0 to 1.

We first investigate whether the model parameters are the same or close when the optimal classification accuracy is achieved over different datasets. The Cauchy model has only one parameter γ, as detailed in Eq. (8). In the subject-dependent decoding, the average accuracy change of all subjects in Dataset 1 (L vs. R) and Dataset 2 is shown in Figure 5 when γ is varied from 0 to 1.

**Figure 6:**
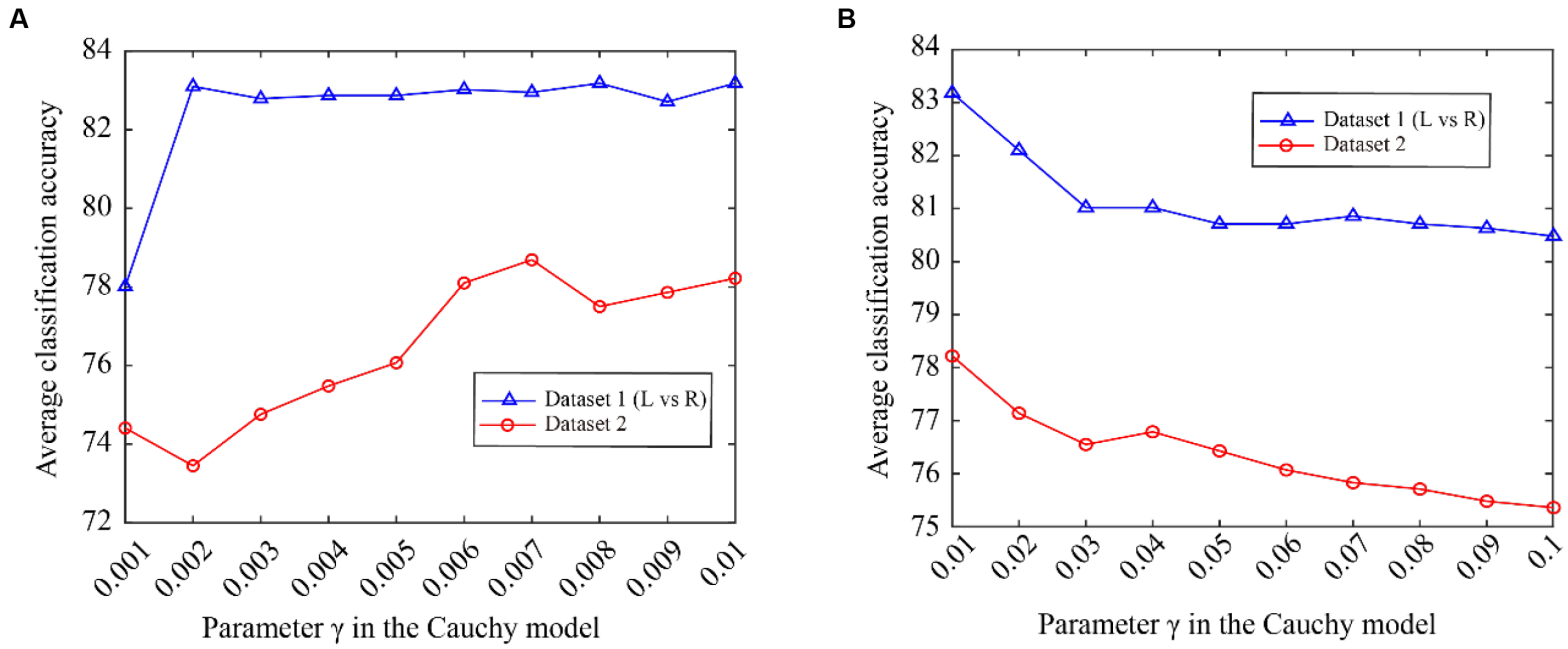
The classification accuracy varies with Cauchy’s model parameter γ. **(A)** ranging from 0.001 to 0.01, **(B)** ranging from 0.01 to 0.1.

From Figure 5, we can see that the classification accuracy change curves of the two datasets are almost the same, indicating that the Cauchy model parameters have good consistency over different datasets. To better represent the consistency of the model parameters, we expand the curves on the fuchsia and lime green boxes of Figure 5, and the results are shown in Figure 6. The fuchsia box part corresponds to Figure 6A, and the lime green box part corresponds to Figure 6B. It can be seen from Figure 6 that the model parameters are also relatively consistent over different data sets, and the parameter values for obtaining the optimal classification accuracy are relatively close.

**Figure 7:**
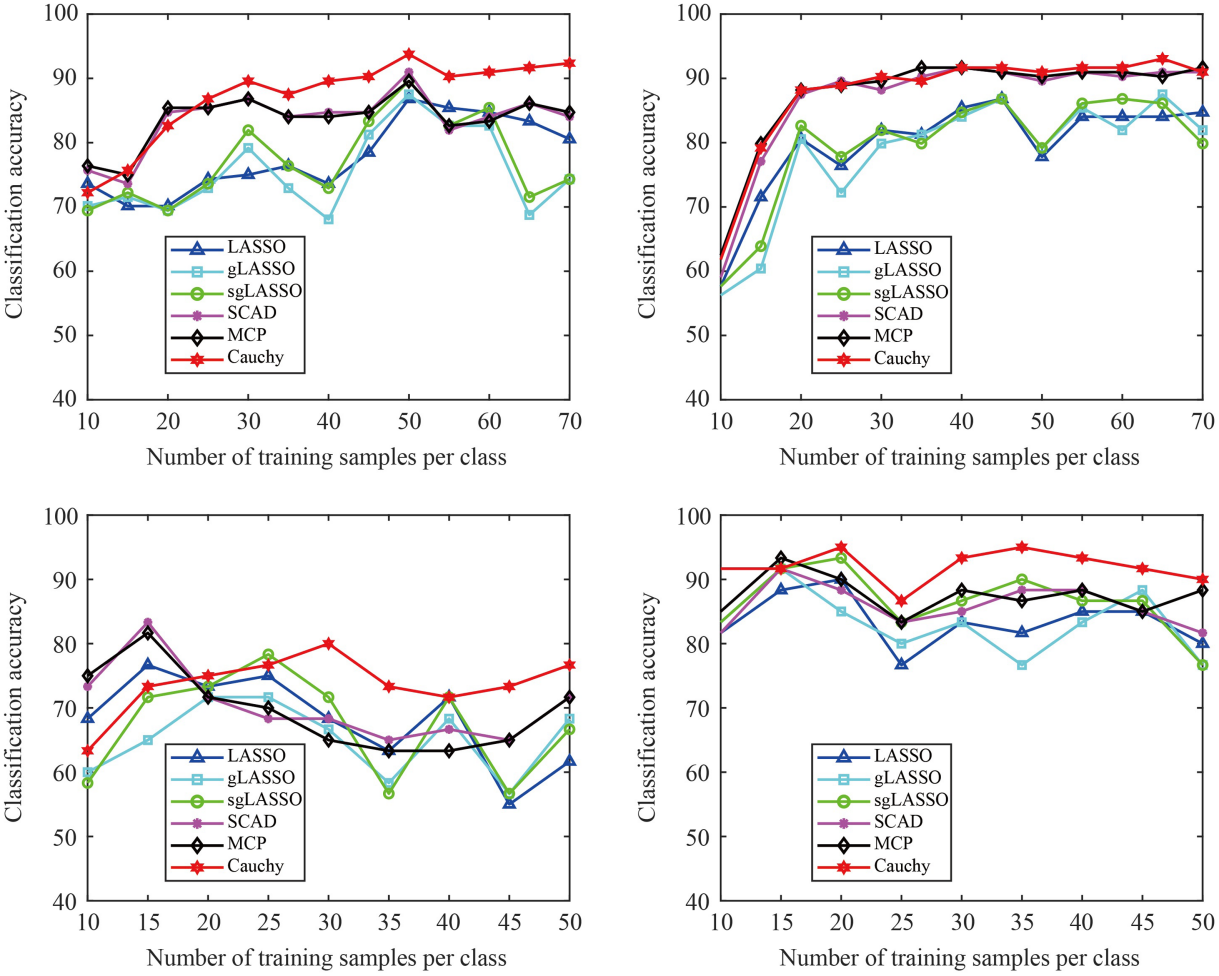
The classification accuracy varies with different training sample sizes. The classification results of four subjects were used for experimental display. **(A)** Subject A01 in Dataset 1 (L Vs R), **(B)** Subject A09 in Dataset 1 (L vs R), **(C)** Subject SO1 in Dataset 2, **(D)** Subject S04 in Dataset 2.

We validated the classification performance of the proposed model across different training sample sizes using the data of subjects A01 and A09 in Dataset 1 (L vs. R) and subjects S01 and S04 in Dataset 2. In Figure 7, the test set remains unchanged, but the sample size of the training set increases sequentially. In addition, the sample size in the training set is the same for both classes of tasks. From Figure 7, we can see that the proposed method is overall superior to existing methods, especially after the training sample size per class reaches 25. Therefore, the proposed method has robust classification ability.

In summary, the proposed model shows better generalization capability with parameter consistency over different datasets and robust classification across different training sample sizes.

## 5. Discussion

We first discuss the overall experimental results, followed by a further analytical study of the feature selection method. Finally, we explore new research directions for future work.

### 5.1. Overall classification results

For the subject-dependent decoding, we can see from Table 2 and Figure 3 that the proposed Cauchy method outperforms the existing feature selection methods. The non-convex regularized feature selection methods (SCAD, MCP, and Cauchy) outperformed the convex regularization methods (LASSO, gLASSO, and sgLASSO), indicating that the introduction of non-convex sparse regularization methods into EEG decoding is effective. Some filtered and wrapped methods also achieve better classification results, but rely on specific classifiers.

For subject-independent decoding, we can see from Table 3 and Figure 4 that the proposed Cauchy method outperforms existing feature selection methods. However, the classification results of existing non-convex regularization methods are lower than those of convex regularization methods. The classification results of most feature selection methods are close and all are low, possibly because the extracted temporal-frequency-spatial features are not distinguishable across subjects. Also, some classifiers work well in subject-dependent decoding, but very poorly in subject-independent decoding. Again, it is shown that filtered and wrapped methods are very much influenced by the classifier.

In addition, we can see from Table 4 that our proposed method works significantly better than the deep learning method in subject-dependent decoding. In subject-independent decoding, although our method outperforms most of the deep learning methods, the classification accuracy of both is not high.

Furthermore, we can see from Figures 5–7 that the proposed Cauchy feature selection method shows better generalization capability.

In summary, the proposed Cauchy method achieved good classification results in both subject-dependent and subject-independent decoding. However, it is still challenging to develop feature extraction and feature selection methods that are effective for both subject-dependent and subject-independent decoding.

### 5.2. Classifier impact on filtered and wrapped feature selection methods

We take F-score and BDE methods as examples to analyze the effect of classifiers on filtered and wrapped methods in subject-dependent and subject-independent decoding. The average classification accuracy is shown in Table 5, which is obtained by averaging the classification accuracies of all subjects in Dataset 1 and Dataset 2, with the maximum value marked with an upper triangle and the minimum value marked with a lower triangle. From the results in Table 5, we can draw two main conclusions. First, the classification accuracies of different classifiers with the same feature selection method vary relatively widely. For BDE in subject-dependent decoding, the maximum classification accuracy is 79.92% and the minimum classification accuracy is 70.43%, a difference of 9.49%. Second, the same classifier with the same feature selection method performs differently on different assessment methods. For F-score, the KNN classifier achieved the best classification results in subject-dependent decoding but was the worst in subject-independent decoding.

**Table 5:** The average classification accuracy achieved by different classifiers with the same feature selection method.

Feature Selection	Assessment Methods	Classifier					Max-Min
		FLDA	BLDA	sBLDA	KNN	LR	
F-Score	Subject-dependent	78.99^▾^	81.42	80.71	82.15^▴^	81.65	3.16
	Subject-independent	62.62	63.44^▴^	63.35	58.15^▾^	62.92	5.29
BDE	Subject-dependent	73.95	79.92^▴^	77.54	79.75	70.43^▾^	9.49
	Subject-independent	63.52	63.98^▴^	63.67	58.19^▾^	63.26	5.79

In summary, the traditional filtered and wrapped methods are influenced by the classifiers. How to select a classifier matching the feature selection method deserves further study. In contrast, the proposed Cauchy method can simultaneously perform feature selection and classification without relying on additional classifiers and thus has a more convenient and efficient performance.

### 5.3. Model analysis for Cauchy feature selection method

The model analysis of the Cauchy method includes model training time and model convergence.

**Figure 8:**
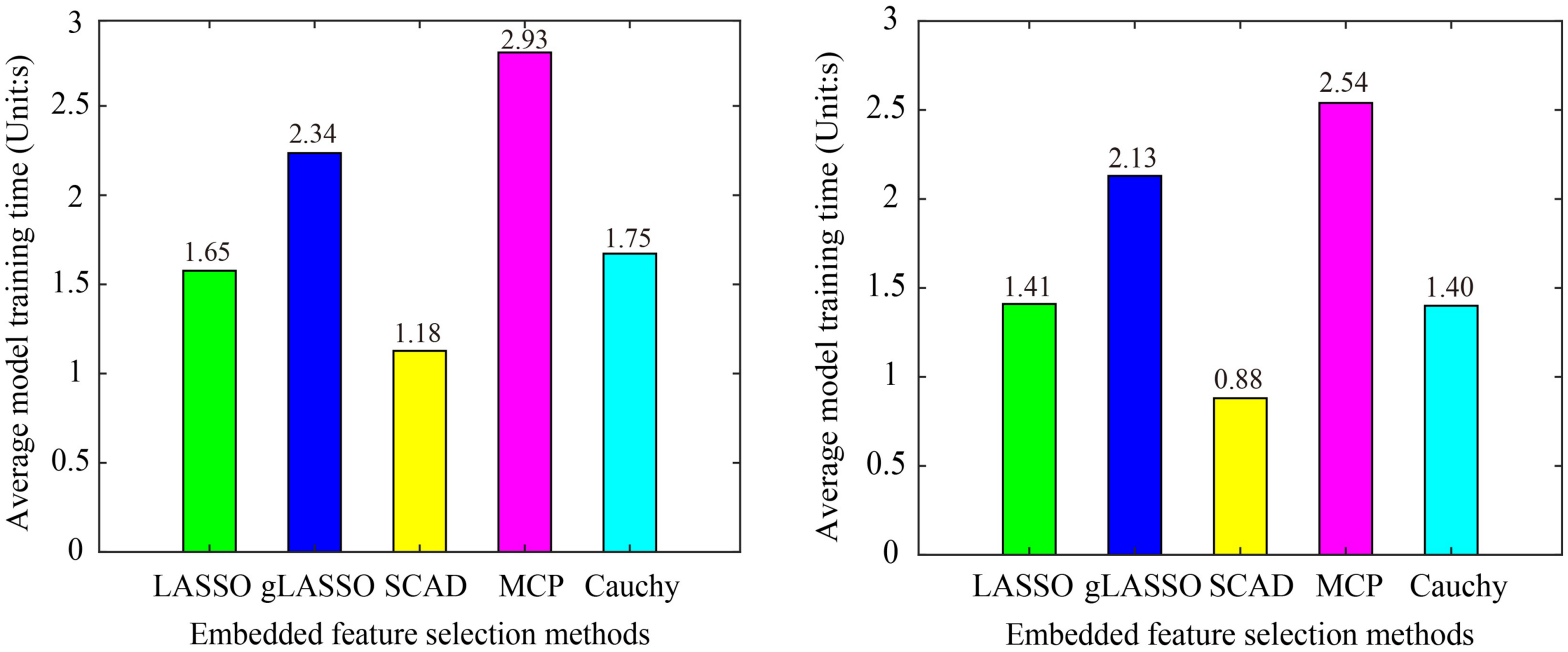
Model training time of various embedded methods in subject-dependent decoding. **(A)** Dataset 1 (L Vs R), **(B)** Dataset 2. The sgLASSO method is not include, because it is model training time is much longer than other methods, which will affect the drawing effect.

We first compared the model training time of six embedded feature selection methods. The program runs in the following environment: OS: Windows 10, CPU: AMD Ryzen 74800H @2.90GHz, RAM: 16GB, MATLAB R2017b. To prevent randomness from affecting a fair comparison, the average model training time of all subjects in the dataset is used as the evaluation criterion. The model training time of the sgLASSO method in Dataset 1 (L vs. R) and Dataset 2 is 130.25 s and 115.11 s, respectively, which is much longer than other methods. To not affect the drawing effect, the sgLASSO method is not included in Figure 8. From Figure 8 we can see that the model training time of the Cauchy method is comparable to LASSO and second only to SCAD.

**Figure 9:**
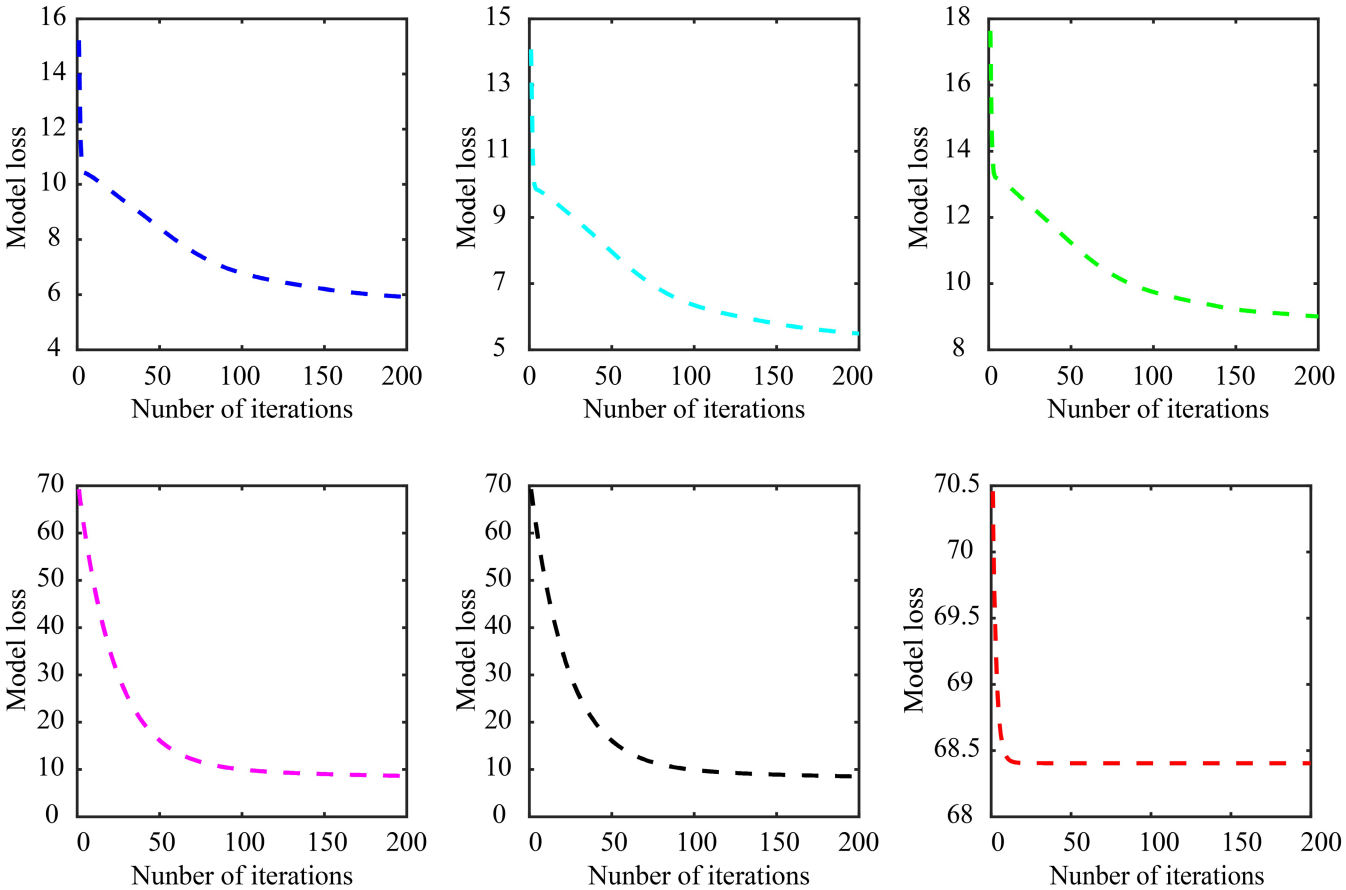
Model convergence curves of various embedded methods in subject-dependent decoding. **(A)** LASSO, **(B)** gLASSO, **(C)** sgLASSO, **(D)** SCAD, **(E)** MCP, **(F)** Cauchy. The regularization parameter λ of the LASSO, gLASSO, SCAD, MCP, and Cauchy methods is set to 2^−2.2^. The inter-group regularization parameter of the sgLASSO method is set to 2^−1^ and the intra-group regularization parameter is set to 2^−2.2^. The model parameter γ of the SCAD, MCP, and Cauchy methods are set to 3, 2, 0.007, respectively.

The model convergence curves of various embedded methods in subject-dependent decoding are shown in Figure 9, still using the data of subject A01 in Dataset 1 for the experiment, in which subject A01 performs left-hand and right-hand tasks. As can be seen, the Cauchy method converges faster and more stable. It is worth noting that the objective function of each feature selection method is different, so the loss range of the model is also different.

In summary, the proposed Cauchy feature selection method has good convergence performance.

### 5.4. Future work

In the follow-up work, we will continue to optimize the solution method of the proposed Cauchy model, such as using the alternating direction multiplier method. In this way, we can improve the convergence speed of the model, reduce the model training time, and make the algorithm more applicable to online brain-computer interface systems.

In this paper, subject-dependent decoding achieves better classification results, but the classification accuracy of subject-independent decoding needs to be improved. We will further explore more effective feature extraction and feature selection methods to enhance the performance of subject-independent decoding. In addition, cross-dataset decoding (Miao et al., 2023; Miao and Zhao, 2023) is also a key direction of our attention.

The extended application of the proposed method is also very important. The proposed method only deals with the data of healthy subjects, and in the future, we will apply it to stroke patients as well as to data from other EEG paradigms, such as P300 and emotional EEG.

## 6. Conclusion

For motor imagery EEG decoding, a non-convex sparse regularization method based on the Cauchy function is proposed in this paper, which can perform feature selection and classification simultaneously, without relying on additional classifiers. The proposed method can effectively alleviate the biased estimation problem of convex sparse regularization models and is closer to unbiased estimation than existing non-convex sparse regularization models. Therefore, the feature selection effect is better than existing methods. The experimental results of the subject-dependent and subject-independent decoding show that the proposed method outperforms existing feature selection methods and deep learning methods. The proposed method shows good parameter consistency over different datasets and robust classification across different training sample sizes. Furthermore, the model training time is shorter and converges faster than existing sparse regularization methods.

## Data availability statement

Publicly available datasets were analyzed in this study. This data can be found at: https://www.bbci.de/competition/iv/, http://bnci-horizon-2020.eu/database/data-sets.

## Ethics statement

The studies involving humans were approved by https://www.bbci.de/competition/iv/, http://bnci-horizon-2020.eu/database/data-sets. The studies were conducted in accordance with the local legislation and institutional requirements. Written informed consent for participation in this study was provided by the participants’ legal guardians/next of kin. The animal study was approved by https://www.bbci.de/competition/iv/, http://bnci-horizon-2020.eu/database/data-sets. The study was conducted in accordance with the local legislation and institutional requirements.

## Author contributions

SZ: Funding acquisition, Methodology, Writing – original draft. QW: Data curation, Methodology, Software, Visualization, Writing – original draft. BZ: Funding acquisition, Investigation, Validation, Writing – review & editing. ZL: Formal analysis, Resources, Validation, Writing – review & editing. LZ: Conceptualization, Data curation, Formal analysis, Validation, Writing – review & editing. LL: Conceptualization, Methodology, Resources, Writing – review & editing. GH: Conceptualization, Funding acquisition, Investigation, Methodology, Supervision, Writing – review & editing. ZZ: Conceptualization, Investigation, Supervision, Writing – review & editing. BF: Conceptualization, Formal analysis, Methodology, Writing – review & editing. TY: Conceptualization, Data curation, Methodology, Software, Writing – review & editing.
